# Dual-Task Performance: Theoretical Analysis and an Event-Coding Account

**DOI:** 10.5334/joc.114

**Published:** 2020-09-29

**Authors:** Bernhard Hommel

**Affiliations:** 1 Cognitive Psychology Unit, Institute for Psychological Research & Leiden Institute for Brain and Cognition, Leiden University, Leiden, NL; 2 Department of Psychology, Shandong Normal University, Jinan, CN

**Keywords:** Action, Cognitive Control, Executive functions

## Abstract

Theorizing on dual- and multi-tasking has not made much progress since the early insight of Telford (1931) and Welford (1952) that response selection may represent a bottleneck in human information processing. A closer look reveals that the questions being asked in dual-task research are not particularly interesting or realistic, and the answers being given lack mechanistic detail. In fact, present theorizing can be considered mere empirical generalization, which has led to merely labeling processing bottlenecks rather than describing how they operate and how they actually produce the bottleneck. As a template for how to overcome this theoretical gap, the Theory of Event Coding (TEC) is applied to dual-task performance. It is shown that TEC, which has not been developed to account for, and has not yet been applied to dual-task performance and its deficits, can nevertheless easily account for the key findings guiding resource and stage theories, while making the underlying mechanisms explicit and transparent. It is thus suggested to consider multitasking costs a mere byproduct of the typical functioning of the cognitive system that needs no dedicated niche theorizing. Rather, what is needed is more mechanistic detail and a more integrative account that can deal with findings related to both resource theory and stage theory.

## Introduction

In stark contrast to many topics studied by cognitive psychologists, the question whether people can perform more than one task at one time has always enjoyed widespread interest. Indeed, technological and societal developments during the past 150 years or so—the time in which systematic, experimental dual-task research unfolded—have strongly increased the complexity of everyday life to a degree that people can hardly manage without at least some degree of multitasking. Accordingly, it does not come as a surprise that many research activities have been devoted to find out whether, and to what degree people can perform multiple tasks concurrently. At the surface, the answer seems obvious: nowadays, few people below 30 seem to manage to walk on the street, ride a bike, take the tram, or steer a car without checking or even working on their smart phone. The more analytical, cognitive view has revealed a more differentiated picture, however. Once tasks are analytically deconstructed into the processes they consist of, it becomes obvious that some processes are more hampered by carrying out other tasks at the same time than others.

Identifying these processes has become the major achievement of academic dual-task research. Since Telford’s (1931) influential studies, it is suspected that response selection is the main culprit, whereas so-called earlier processes, like feature extraction or perceptual identification, and so-called later processes, like action execution, seem to suffer much less or not at all from multitasking (e.g., Pashler, 1994). Hence, the hypothetical processing bottleneck is commonly identified with response selection (Welford, 1952; Pashler, 1984). Others were even more reluctant to explicitly refer to a particular process or system, and suggested that there may be a general resource that is limited, but which process is affected by that may depend on the resource-allocation policy of the individual (e.g., Kahneman, 1973; Tombu & Jolicoeur, 2003). All these suggestions have been challenged both theoretically and empirically (e.g., Navon, 1984), and there is currently not a single approach that would be able to tackle all the challenges. For instance, even the rather general assumption of a single channel, which according to Welford (1967, p. 5) “holds that the central decision mechanism can deal with data from only one signal, or group of signals, at a time, so that data from a signal arriving during the reaction time to a previous signal have to wait until the decision mechanism becomes free” has been empirically disconfirmed (e.g., Hommel, 1998). Along the same lines, the idea of response selection as the sole bottleneck does not quite fit with empirical demonstrations that response selection in one task can interfere with memory retrieval (Carrier & Pashler, 1995) or working memory encoding/update (Jolicoeur & Dell’Acqua, 1998) in another. The response-selection account has been tried to be salvaged by assuming that response selection can also be considered memory retrieval, which however runs into the problem that concurrent retrieval of responses can rather easily be empirically demonstrated (Hommel, 1998). So, taking altogether, it is fair to say that there is no established, widely accepted model that explains when, how, and why carrying out more than one task hampers performance. We do know *that* performance can suffer, but we don’t know *why*, which is a rather disappointing resume after more than 100 years of academic research on this topic. In my following analysis, I try to understand why that is the case and how the situation could be improved.

## What is the question?

As mentioned already, the often-posed question whether people can carry out more than one task at a time can be answered affirmatively even without any scientific evidence: we do that every day. The scientifically more interesting question is whether we can do so without costs, be they short-term, such as when one task is performed less well in the presence of another, or long-term, such as when multitasking exhausts the agent more than single-tasking. While the latter question is rarely posed, the former has enjoyed substantial attention. However, the available experimental demonstrations of multitasking costs do not quite fit with observations from everyday life: jugglers, piano players, drummers, basketball players, and others clearly carry out more than one task at one time without any visible costs. This does not rule out the possibility that systematic measurements of high precision could reveal possible costs at a millisecond level, say. It also does not rule out the possibility that seamless performance was achieved by reconfiguring possible bottleneck processes in such a way that they no longer interfere with each other (Ruthruff, Johnston & Van Selst, 2001). But it does mean that academic research into multitasking, and the findings it has generated so far, have very little, if any meaning for real-life performance (which is not meant to underestimate the societal importance of empirically demonstrating objective costs of multitasking, e.g., while driving: Levy, Pashler, & Boer, 2006).

A related criticism has been put forward by Miller, Ulrich, and Rolke (2009), who used mathematical simulations to find out whether true multitasking, if it would be possible, would actually represent an advantage over single tasking. According to their assessment, multitasking would be more efficient than single tasking under only very few, very special circumstances. This undermines the common intuition that multitasking would be the ideal case to carry out tasks, so that empirical observations of single tasking would count as a deficit and imply a structural, unsurmountable bottleneck. A similar case was made by Meyer and Kieras (1997), who considered multitasking a strategic choice that agents may or may not prefer over single tasking.

While these considerations imply that most of the theoretical discussions in the domain of multitasking bear little, if any relevance for understanding everyday behavior, one could still argue that the question whether some processes can be carried out in parallel while others cannot is of scientific relevance (Townsend, 1990). Indeed, the question whether cognitive processes run serially or in parallel has been occupied psychological research for decades. However, this endeavor seems to rely on the careful definition and analysis of the process in question, which stands in stark contrast with the coarse-grained and rather anti-theoretical labeling strategy applied by dual-task researchers. Testing for serial versus parallel processing should rely on specific hypotheses that are based on mechanistic insight into the processes under task, but instead of developing such hypotheses, dual-task researchers have relied on methodologies like the locus-of-slack logic (Pashler, 1994) to generate theoretical insights in an entirely data-driven fashion.

## What are the answers?

Even though close consideration reveals that the questions underlying theorizing about dual- and multitasking are opaque and indeed questionable, it may be that the answers are more convincing. So, let us now turn to the answers that research on multitasking has given. There are in fact two rather different types of answers.

### Resource accounts

One is related to the concept of a capacity-limited resource, as in the seminal approach of Kahneman (1973) or later approaches by Navon and Gopher (1979) or Tombu and Jolicoeur (2003). The idea is that there is some sort of mental energy that the processes responsible for carrying out a task are in need of. The more resources a process receives, the better and the more efficient it operates. However, as the resources are limited, increasing the number of processes implies that each process receives fewer resources, so that adding a second or more tasks, and all the processes responsible for carrying them out, is likely to overload the system. Whereas a little overload may be compensated by smart allocation policies, increasing overload will at some point lead to measurable performance costs.

Resource models have been criticized on several grounds, and studies have revealed that the assumption of one homogeneous pool of energy cannot explain why deficits are often relatively process-specific (cf., Sanders, 1997). This has led to the formulation of more process-specific resources (Navon & Gopher, 1979; Wickens, 1984) or of resources that affect the degree of parallel processing of particular stages (Tombu & Jolicoeur, 2003), which has effectively diminished the conceptual discrepancies between resource models on the one hand and the processing-stage models to be discussed below on the other. But my main concern is of a different nature. Let us assume, just for the sake of the argument, that the original resource models had not been empirically and conceptually challenged, and that computational versions would be able to make predictions that could be empirically confirmed (as in Tombu and Jolicoeur, 2003). Of what kind would be the answer that such models provide, and what would we learn from that answer?

The most obvious question arising from this question concerns the nature of the hypothetical energy or resource: What is it? What is it made of? The disappointing answer is that we still don’t know, it has remained purely hypothetical and neither functional nor neural analyses have managed to bring any light into this matter. Particularly worrying is the fact that this is not because interesting hypotheses were put forward but empirically disproved. Rather, it is because no one was searching. Closest to a possible solution were assumptions that likened the hypothetical resource to arousal, or effort, or both (e.g., Kahneman, 1973), but given that these concepts were equally undefined as the resource itself, this could hardly be accepted as a satisfying solution. Without any idea what the hypothetical resource might represent, corresponding approaches get worryingly close to animistic theorizing, which accounts for the behavior of objects or agents by attributing to them some spiritual essence that has no other purpose than producing that behavior. The same criticism applies to the allocation of resources, which in resource models is essential, because it does almost all of the explanation. Again, almost nothing is known about how people allocate their resources, which cues they are using to do that, and how the allocation is actually achieved. What are the mechanisms sensing the need for more resources devoted to a particular process or stage, and what is the machinery that reroutes the mysterious resources to target them? Again, there are large gaps in our knowledge and not even ideas how to reduce them in principle.

This doesn’t mean that the assumption of some capacity-limited resource is necessarily incorrect. It may well be that there is some limitation with respect to, for instance, overall activation of the brain or relevant brain areas (e.g., due to the dependency of this activation on oxygen and blood flow), sugar needed to drive the frontal lobe (Gailliot et al., 2007), or bandwidth limitations of neural synchronization, or of the functional processes relying thereupon. However, hardly any attempts were made to test such possible links or to put forward other kinds of hypotheses. This means that the resource approach may allow for the successful prediction of a number of empirical phenomena and their dependency on, or sensitivity to particular experimental manipulations, but it fails to provide the essence of a cognitive approach: a mechanism that would allow us to really understand multitasking limitations. In fact, assuming a capacity limitation provides only little more than a redescription of the findings. After all, what needs to be explained is a limitation of the capacity of people to perform multiple tasks at the same time, so that hypothesizing some limited capacity to account for that is very close to circular reasoning.

### Stage accounts

The key competitor of resource accounts is the processing-stage account. The basic idea underlying it is that human performance emerges from information processing that runs through several stages, from early perceptual encoding to motor execution, and the main question then is which of these stages allow for parallel processing and which for only serial processing (Sternberg, 1969). Clever experimental designs were developed to tell serial from parallel stages (Pashler, 1984) and the bulk of the evidence points to one single stage: response selection (Pashler, 1994). As I have mentioned above, there are a few observations that also imply some limitations regarding memory retrieval, and some ideas how they may fit with the response selection account (e.g., Pashler & Johnston, 1998). While these ideas might be perfectly correct, they point to a very general problem of the stage account: there is simply no theoretical constraint or guidance with respect to the kind and number of stages involved in information processing, or the order in which they are thought to operate.

Given that stage theory is based on the idea of a rather rigid sequence of serially operating stages, this is particularly worrying if one tries to relate two or more models to each other or integrate models of different effects or phenomena. If, as Pashler and Johnston (1998) suggest, memory retrieval is indeed an operation involved in response selection, how can one model this in terms of stage theory? Can one equate response selection with memory retrieval? Under which conditions? Does this hold for all kinds of memory? Or how do we account for effects of the second task in a dual-task paradigm on the first (Hommel, 1998, see below)? Stage theorists might respond to these challenges by drawing lines from the boxes representing secondary-task stages to the boxes representing primary-task stages, but is that really more than a redescription of the empirical findings?

However, my main concern with stage accounts is of a somewhat different, even more fundamental nature. Let us thus assume for a moment, again just for the sake of the argument, that it is only the response selection stage that is the culprit, whereas stages responsible for stimulus feature extraction, identification, and response execution allow for perfectly parallel processing. What do we learn from that? Does that give us an idea why this bottleneck exists? What is so different about selecting a response that it hampers multitasking so much more than selecting a stimulus, say? Which characteristic of the response-selection process helps us to understand why it puts so severe limitations on our multitasking abilities? Similar to resource theorists, stage theorists have nothing to offer and did not even make attempts to address these questions. Why is that?

## Lack of mechanistic theorizing

As elaborated elsewhere (Hommel, in press), the answer is likely to do with what Lewin (1931) has called an Aristotelian approach to psychology. In physics, theoretical approaches commonly apply the research strategy that Hempel and Oppenheim (1948) have called “explanation as subsumption under natural law.” The general idea is that empirical phenomena are scrutinized for shared systematic features. If the sharing of these features is sufficient, they are grouped together and assumed to embody a natural law, such as Newton’s law of motion. Theorizing in psychology often tries to mimic this practice by effectively equating natural laws with categories of observations (Cummins, 2010)—an exercise of “empirical generalization” (Fiedler, 1991).

If, thus, irrelevant response-incongruent color words hamper performance in a color-naming task, irrelevant response-incongruent letter flankers hamper performance in a letter-identification task, and irrelevant response-incongruent locations hamper performance in a color-identification task, cognitive psychologists take the commonalities between these observations to generalize that irrelevant response-incongruent stimuli tend to hamper performance. This generalization is than commonly treated similar to laws in physics and taken as a given, that is, taken as a sufficient explanation for the observations. One may encounter that “mechanistic models” have been developed to account for the observations, such as the dimensional overlap model (Kornblum, Hasbroucq & Osman, 1990). However, the theoretically most crucial question of why irrelevant stimuli trigger responses they are associated with is answered by an arrow originating in the stimulus box and pointing to the response box—which is a valid redescription of the findings and the empirical generalization it has motivated, but not really an explanation that would help us to understand *why* and *how* irrelevant stimuli are doing this. With respect to dual-task performance, the same has happened: given that all manipulations that involved some variation of response-selection in the primary task have increased the dual-task costs on the secondary task, a response-selection box was invented and taken as an explanation.

In practice, this translates into the explaining-through-categorizing strategy that Lewin (1931) considers characteristic of an Aristotelian approach. This strategy shares many features with evolution biology, which assumes that there is a limited number of natural kinds, species that can be organized by means of hierarchical taxonomies. Whenever a seemingly novel animal is discovered, the scientific challenge consists in sorting, that is, in determining whether the given animal actually belongs to a species that is already known or whether it requires the assumption of a new species that has then to be located in a slightly reorganized taxonomy. Along the same lines, stage theorists see their challenge in organizing empirical phenomena into taxonomies, commonly consisting of series of hypothetical processing stages, so that each seemingly new phenomenon, such as an effect or observation, can be assigned to a particular stage.

As Lewin (1931) has pointed out, such an Aristotelian approach might be necessary in the beginning of a science but it quickly runs into limitations. Most importantly, and that becomes obvious in the case of stage theorizing, considering sorting as an endpoint of the theorizing process provides no insight into the actual mechanism. While stages are labeled according to a particular function, such as response selection, no attempt is being made to look inside the stage, to understand how the labeled process actually operates. According to Lewin, understanding how it operates can only be achieved through what he calls a Galilean approach, which requires theories that resolve previous categories into continua, and that account for both group differences and individual variability by means of the exact same model or theory. The strong integrative power of such a Galilean approach is obvious, and Lewin’s plea for such an approach in a certain sense anticipates the later complaint of Newell (1973), that “you can’t play 20 questions with nature and win”. Applied to dual-task performance, such an approach would require a much more precise understanding of what mental resources might stand for, how and according to which criteria resources are allocated, how the assumed processing stages actually operate, and how all that explains when, how, and why our multitasking abilities are limited.

Taken altogether, we need more mechanistic theorizing to really understand the difficulties of humans to carry out more than one task at a time. Even though the term “mechanism” features in the majority of the respective literature, I’m not aware of any attempt to actually provide a concrete mechanism. As elaborated elsewhere (Hommel, in press, p. 1; cf., Bechtel, 2008, 2009), a truly mechanistic model or theory of cognitive phenomena needs to “consist of a clear specification of its components, such as the codes or representations of the relevant informational units, and of the organization of these components, including the processes operating on them” or, in short, it needs to explain how structures relate to processes, and vice versa. What resources stage models do is to label the processes, thereby skipping the structures or codes on which they operate, and fail to provide us with any insight into how the processes operate in a way that would allow us to really understand why multitasking is so difficult. Given that many approaches that do try to provide more detail about processes like stimulus identification or response selection (as featured in stage models), or about individual strategies to adjust to situational circumstances (as implied by resource models), this gap in our understanding is unlikely to do with the state-of-the-art of our discipline in general. Rather, it seems to be a lack of ambition to dig deeper, presumably due to the widely shared Aristotelian attitude that still characterizes cognitive psychology, even about 90 years after Lewin’s suggestion to move forward.

On the one hand, this is a disappointing diagnose, because it means that our field could be much more advanced than it currently is. On the other hand, however, it means that there is no structural limitation that prevents us from moving forward. We can actually begin immediately by asking more mechanistic questions and working on more mechanistic answers. Ideally, we do this not by piecemeal modeling, that is, by constructing a different model for each of the highly artificial effects that research on multitasking has generated but, rather, by using a single framework to integrate, step-by-step, all the phenomenon that we consider theoretically relevant. To end on a more constructive note, I have tried to sketch a possible approach in the following. Given my own background on perception and action, I chose the Theory of Event Coding (TEC: Hommel, Müsseler, Aschersleben & Prinz, 2001) as a point of departure—the arguably most comprehensive theory of human perception, cognition, and action. I would like to emphasize that this theoretical framework was originally developed to account for phenomena that are entirely unrelated to multitasking, and with a rather strong emphasis on representations (as a counter-reaction to the previously rather exclusive focus on processing stages). However, the framework has been developed over the years to become more balanced with respect to both representations and processes, to become more explicit with respect to the questions how the interactions between representations and processes is controlled by tasks and goals, among other things, suggesting that it may be of rather general use for various purposes (Hommel, 2009, 2018, 2019; Hommel & Wiers, 2017). This does not necessarily make TEC the only or the best framework for guiding our theorizing towards a better understanding of the mechanisms underlying dual-task performance, and I do not even claim that it is particularly suitable. The only claim I want to make is that connecting findings and insights from research on multitasking to models and theories that are more explicit with respect to representations and processes involved in human information processing is likely to increase our understanding of multitasking limitations and to allow for more, more novel, more interesting, and more diagnostic hypotheses the empirical testing of which might lift research on dual-task performance to the next level.

## An event-coding approach to dual-task performance

**Figure 1:**
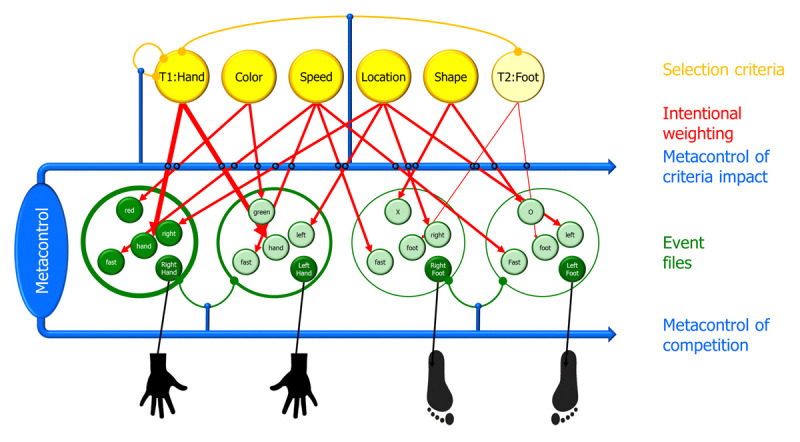
Schema illustrating the application of the Theory of Event Coding to dual-task performance. Task 1 is assumed to require a left- or right-hand response to the green or red color of a stimulus, and Task 2 is assumed to require a left- or right-foot response to the letters O or X, respectively. The possible responses are represented by bindings of sensorimotor features (event files) that compete for action control (indicated by mutually inhibitory connections). The example shows only those features that are relevant for the tasks and that (therefore) receive intentional weighting from selection criteria. Goals consist of selection criteria that operate through intentional weighting (i.e., by increasing the weights of task-relevant dimensions). These are matched against the features included in all available event files, inducing stronger activation of criterial-matching feature codes and, as a consequence, of the event file containing them. The impact of criteria on the activation of event files and the degree of competition between event files is modulated by metacontrol, which varies between high persistence and high flexibility, maximizing or minimizing impact and competition, respectively.

TEC has a strong ideomotor flavor, as reflected in the assumption that the basic units of human cognition and action planning consist of bindings of motor patterns, represented in Figure 1 by the codes that are directly connected to effectors (left and right hands and feet in this example) and codes representing the perceptual consequences of performing this action, acquired through previous sensorimotor exploration (codes HAND, FOOT, LEFT, and RIGHT in the example). Each binding, or event file (Hommel, 2004), also includes codes representing other features like action features emphasized in the instruction (FAST, in the example) and the context in which actions are or should be carried out (here: the color or shape of the respective stimulus).^1^ Event files of that sort have been empirically shown to be constructed during the task instruction and maintained from thereon (Cohen-Kdoshay & Meiran, 2009). Event files are assumed to compete with each other, as indicated by the inhibitory links between the two hand reactions and the two foot reactions (inhibitory links between hand and foot reactions are also likely to exist but not sufficiently active to play a role here, for reasons explained below). The activation of an event file depends on several conditions and comes from several sources. First, only actively prepared event files play a recognizable role. Here, I have assumed that only event files representing instructed actions have been sufficiently prepared. Second, any internal or external signal or stimulus that activates one or more codes representing its features will tend to activate the event file it is a part of. Accordingly, presenting a red stimulus is likely to activate the right-hand action. The impact of processed features on code activation is moderated by intentional weighting (Memelink & Hommel, 2013), which is controlled through selection criteria that together can be considered to represent the current goal (Hommel & Wiers, 2017). If, for instance, the codes representing the criteria hand, color, fast, location, and shape are active, as in the figure, features falling on the perceptual dimensions that the criteria indicate will receive a higher output again, meaning that they have a stronger impact on activating event files.

Figure 1 is aimed to capture the essence of a dual-task situation, in which Task 1 requires left and right hand responses to green and red stimuli, respectively, and Task 2 left and right foot responses to the letters O and X, respectively. The four depicted event files represent the four acceptable and thus prepared actions in this task situation. The activated selection criteria define all the perceptual dimensions that are relevant in the task: hand and foot by defining the effectors being involved, color by defining the stimulus for Task 1, speed because this is assumed to be emphasized in the instruction, location by defining the alternative responses for each response effectors set, and shape by defining the stimulus for Task 2. Depending on their degree of activation, criteria will boost the processing of features falling onto the dimensions they specify. Here I have assumed that the instruction requires the response to the first task to be carried out before the response to the second. This is represented by stronger activation of the hand criterion than the foot criterion. As soon as Task 1 is completed and the hand action is carried out, the corresponding hand criterion is assumed to inhibit itself, which reduces its inhibitory impact on the foot criterion, so that this criterion will gain in activation and contribute to the selection of the response to Task 2 (borrowing the mechanism of serial action control from Rumelhart & Norman, 1982).

A final feature of recent versions of TEC comes under the label of metacontrol. Various authors have emphasized that truly adaptive action control should be able to adopt different processing styles. Under some circumstances, like in a Stroop task, it may be particularly useful to focus on information that is task-relevant but ignore all distractors, while under other circumstances, like in a brainstorming session, information processing should be much less restricted but more integrative and associative. This general idea has been discussed under various labels, like persistence or stability vs. flexibility (e.g., Dreisbach & Goschke, 2004) and exploitation vs. exploration (Cohen, McClure & Yu, 2007), and related to underlying brain mechanisms, which presumably include the dopaminergic pathways (for a review, see Hommel & Colzato, 2017a). According to Hommel (2015; Hommel & Colzato, 2017a), this kind of adaptivity can be captured by assuming that metacontrol operates by modulating the degree to which alternative decision alternatives compete in the degree to which action goals are biasing the competition between these alternatives. Following Hommel and Wiers (2017), Figure 1 shows metacontrol to modulate intentional weighting (i.e., the degree to which selection criteria boost the impact of feature codes on activating the event files they belong to; see *Metacontrol of criteria impact*) and the strength of mutual inhibition between event files (see *Metacontrol of competition*).

### Accounting for findings thought to support resource theory

Let us know apply this architecture and the example configuration to some of the key findings of research on dual-task performance. Let us begin with two theoretically particularly diagnostic findings from resource theory. One consists in the well-replicated observation that instructing individuals to emphasize one of two tasks more than the other has a corresponding impact on relative performance: the higher the emphasis the smaller the dual-task costs in this task and the larger the dual-task costs in the other (for an overview, see Sanders, 1997). In TEC, instructions of that kind are most likely to affect either intentional weighting or metacontrol, or both. In this particular case, an impact through metacontrol is the most plausible alternative. Emphasizing one task over another is likely to shift metacontrol towards more persistence, not unlike the instruction used to have individuals engage in focused-attention meditation (Hommel & Colzato, 2017b). In the example, this would translate into stronger competition between event files and, more importantly, between selection criteria. If, thus, Task 1 would be emphasized over Task 2, as the typical instruction to carry out Task 2 not before completion of Task 1 in a sense already implies, the mutual inhibition between the hand and the foot criterion would become larger. If so, switching from the first to the second task would take longer and, as a consequence, performance in the second task would suffer. Moreover, due to the stronger mutual competition between the two criteria, the relative dominance of the hand criterion would increase and thus speed up the selection of hand actions, which in turn implies that Task 1 performance gets better.

Another diagnostic finding predicted by more recent versions of resource theory (Tombu & Jolicoeur, 2003) is that processing can vary in the degree of seriality and parallelism. In TEC, this can also be achieved, but in ways that are entirely unrelated to any kind of resource. Again, shifting metacontrol towards the persistence pole would make information processing more serial (due to the increased mutual competition between alternative responses) while shifting it towards the flexibility pole would make it more parallel (due to the reduced mutual competition). Two findings confirm and illustrate this expectation. Both used the so-called backward-compatibility effect (BCE) first reported by Hommel (1998). The effect consists in the observation that performance in the first task is affected by the compatibility between stimulus or response features in the first and the second task (e.g., a left response in Task 1 is initiated faster if Task 2 also requires a left response; cf. Logan & Schulkind, 2000). This observation suggests that, in the case of response-response compatibility, the second response has already been activated before the selection of the first response was completed, which makes the BCE an indicator of the degree of distributed, parallel processing (Logan & Gordon, 2001; Lien & Proctor, 2002). Importantly for our purposes, Fischer and Hommel (2012) obtained a smaller BCE if participants were primed with a convergent-thinking rather than a divergent-thinking task. Given the evidence that engaging in convergent thinking shifts metacontrol towards the persistence pole (Hommel & Colzato, 2017a), this implies that more persistent metacontrol promotes serial processing. Relatedly, Hommel et al. (2016) found a larger BCE after exposing participants to binaural beats in the gamma range, which was assumed to bias metacontrol towards more flexibility.

Admittedly, more, and more specific research would be necessary to further strengthen the link between metacontrol and fluctuations in task emphasis and the degree of parallel processing. Can different “stages” be selectively affected? Can metacontrol become conditioned to external cues that regulate the balance between persistence and flexibility? Which cues do people use under natural circumstances? Importantly, however, none of these questions and, presumably, none of the resulting answers implies some mysterious, not further specified resource or set of resources, nor provides resource theorizing obvious accounts for the findings of Fischer and Hommel (2012) or Hommel et al. (2016). Instead, TEC provides a rather transparent cognitive architecture comprising of representations, processes, and specific assumptions about how the interaction between representations and processes generates empirical observations—in stark contrast to resource accounts that, with few exceptions (Wickens, 2002), provide none of that.

### Accounting for findings thought to support stage theory

Stage accounts of dual-tasking costs, and response-selection-stage accounts in particular, are based on four findings that are taken to be particularly diagnostic (Pashler, 1994). The basic idea is that Task 1 and Task 2 require running through a series of stages, in which some can be run through in parallel while others cannot. Given that the stages that cannot be run through in parallel are considered to indicate the bottleneck underlying dual-task costs, identifying those stages is key, and having identified those stages is considered to provide a complete explanation of dual-tasking costs. This account makes four particularly diagnostic predictions. P#1 is that prolonging the bottleneck stage(s) in Task 1 should delay the responses of both tasks equally; P#2 is that prolonging the post-bottleneck stage(s) in Task 1 should not affect Task 2; P#3 is that prolonging the pre-bottleneck stage(s) in Task 2 (at least within a certain range) should not delay any of the two responses; and P#4 is that prolonging the Task-2 bottleneck stage should delay the response in Task 2 but not in Task 1 (see Pashler, 1994). While not all of these four hypotheses have enjoyed equally intensive testing, most of the available evidence doing so points to response-selection as the sole bottleneck stage (Pashler, 1994; Pashler & Johnston, 1989).

Let us now see whether TEC can deal with these findings. P#1 actually consists of two parts, of which one is trivial: if the response of Task 1 would not be delayed by the experimental manipulation, this manipulation would not have been successful and the prediction would thus no longer hold. The second part is more interesting, namely, whether a successful manipulation would also delay the response of Task 2. In the configuration shown in Figure 1, this part of the prediction directly follows from the implementation of the instruction: given that participants are requested to begin with Task 2 not before the completion of Task 1, the foot code that the model assumes to initiate the actual selection process receives the necessary boost of activation only after the selection controlled by the hand code has been finished. For one, this means that it is the completion of response selection that counts in TEC, which provides a natural account of the fact that response selection is assumed to be the bottleneck (for a very similar reasoning, see Logan & Gordon, 2001). For another, this implies that it is the completion of the response selection in Task 2 that is delayed, whereas the codes included in the event files related to Task 2 can become activated before response selection in Task 2 has “officially” started. This provides a natural account of the BCE discussed above: if the stimulus for Task 2 appears before Task-1 response selection has been completed, it can already prime the corresponding event file, which activates the corresponding codes of this file to some degree, so that they can “backward-prime” the same codes involved in other files. Hence, TEC provides a natural implementation of the distinction between (parallel) stimulus-response translation and (serial) response selection suggested by Hommel (1998).

Evidence supporting P#2 is also perfectly compatible with the TEC model. If we consider response selection as the bottleneck, the time demands of everything following response selection (which commonly goes under the label of response execution) in Task 1 should not affect performance in Task 2. On the one hand, TEC does not assume that response selection is a discrete act, which means that even responses that have been launched already can be still affected and even shaped by sufficiently activated codes—an assumption that has received empirical support (Hommel, 1996; Hommel, Lippelt, Gurbuz & Pfister, 2017). On the other hand, however, this has no bearing on predictions of dual-task costs and should not affect the time point of shifting activation from the hand to the foot code in our example scenario. Accordingly, TEC is fully compatible with findings supporting P#2.

P#3 is commonly considered to be the most surprising prediction from the stage approach. It has therefore been frequently tested and has received ample support. This suggests that making Task-2 stimulus processing more difficult should not delay either response, the idea being that Task-2 response selection has to wait for the completion of Task-1 response selection anyway, so that the greater difficulty in processing the Task-2 stimulus should no longer be visible in the reaction times. Again, TEC provides a natural account of findings supporting this prediction: the model is actually fully parallel, it is only the implemented instruction that makes response selection serial (exactly as suggested by Meyer and Kieras, 1997). Accordingly, the activation of stimulus codes related to Task-2 event files can begin as soon the corresponding stimulus appears, so that a delay in the activation of this code would not be visible in the time it takes to select the Task-2 response. Thus, TEC is fully compatible with findings supporting P#3.

The same holds for findings supporting P#4, which again consists of a trivial and a non-trivial part. The trivial part relates to Task 2: if performance in this task would not be measurably affected, the corresponding manipulation would have failed. The nontrivial part relates to Task 1, which should not be affected by the manipulation of response-selection difficulty in Task 2. Again, TEC is fully compatible with findings supporting this prediction, as it would not provide any no reason why delaying response selection in Task 2 should have any impact on Task 1. Hence, taken altogether, TEC cannot only fully compete with the classical stage-theoretical accounts of dual-task costs, but it also provides natural explanations for observations that stage accounts have difficulties with, such as the BCE.

## Conclusions

The argument that I have developed here is that present theorizing about dual-tasking is under-ambitious and lacks mechanistic depth. As I have tried to explain, this is because of the widespread acceptance of more or less descriptive models that go hardly beyond empirical generalization and provide no insight into the actual mechanisms, that is, into the representations involved, the processes that operate on these representations, and the particular way that these interactions generate the phenomenon under investigation. This has led to the unfortunate situation that our current insight into the underpinnings of multitasking hardly go beyond the considerations of Telford (1931) and Welford (1952), quite some decades later. My suggestion how this situation can be improved is to stop playing 20 questions with nature and lose (Newell, 1973) and turn the current Aristotelian approach into a Galilean (in terms of Lewin, 1931). Among other things, such an endeavor would be likely to benefit from the following subgoals.

First, it seems essential to develop more mechanistic models and theories of dual- and multi-tasking. Simply labeling processes should not be enough, what we need is to look inside the boxes of current stage theories and explain what is going on there and how this is achieved in terms of clearly specified processes operating on clearly specified representations. In the previous section, I have tried to develop a template of how this might look like, by using the cognitive infrastructure provided by TEC. One may prefer other cognitive infrastructures or a different level of modeling (e.g., neural networks), but every attempt to go beyond empirical generalization would be welcome and important.^2^

Second, it does not seem to be necessary to develop tailor-made dual-tasking models. In the previous section, I have used TEC as a point of departure, an infrastructure that was not developed to account for multitasking and that has not yet been considered in this context. The fact that it nevertheless can easily compete with the well-established, traditional resource and stage theories of dual tasking implies that multitasking costs may not need any special, dedicated theorizing but might be a mere byproduct of how our cognitive system operates. Using more general theories with a wider scope and much more independent empirical support to account for multitasking and derive new hypotheses has the additional advantage of easily relating dual-task paradigms to other psychological phenomena that these theories can also account for. Moreover, using these theories would turn dual-task studies into additional empirical tests of them, which may further test the validity of these theories and in some cases suggest further improvements. Hence, replacing the current niche theories by broader theoretical frameworks would be likely to create a win-win situation.

Third, Galilean psychology would imply more theoretical consideration for individual differences. While the Aristotelian approach commonly considers both inter- and intra-individual variability as mere noise,^3^ as reflected in the fact that our statistical procedures are interested in mean differences only, the Galilean approach would require accounting for each individual at each time, at least in principle. However, accounting for individual differences also requires a more mechanistic account of multitasking that provides the necessary parameters according to which individuals might differ. At the same time, an eye and more appreciation for individual differences would be likely to make better use of participants, as the variability of their performance may no longer need to be overcome by increasing power but provide a welcome opportunity to test one’s theory.

Finally, it is interesting to see that key findings from both resource theory and stage theory can be easily modeled by the same general account, suggesting that these two theoretical approaches may be less incompatible than previously assumed. Moreover, TEC has the advantage of accounting for resource-like effects without the need to postulate any mysterious, not-further-specified energy and with a much clearer specification of how and why response selection might represent a bottleneck. Clearly, the present suggestions are very preliminary, and I do not pretend to provide a full-fledged model of multitasking already. In fact, the TEC approach raises many new questions that call for empirical study: Can event files be integrated across tasks, e.g., through practice? Can multitasking costs be eliminated under suitable conditions? Will the increase of feature crosstalk increase persistence? And yet, given that a few trivial adjustments were sufficient to make TEC a strong competitor of classical models suggest that developing a full-fledged TEC model of multitasking should not be too difficult.

## Data Availability

This makes little sense for a theoretical paper, as no data are reported. Comparable theoretical papers of your journal have also no such a section.

## Footnotes

Note that the figure is strongly simplified. For instance, the shape of Task-1 stimuli and the color of Task-2 stimuli, which are also likely to be represented, are omitted, as are features of the particular movement being carried out and more.

One may argue that even a TEC approach is sorting phenomena, e.g., by “subsuming” various multitasking costs under the “principle of feature binding”. While this is semantically defendable, this kind of sorting would be based on similarities regarding the postulated underlying *mechanisms* (i.e., the explanans), whereas the sorting I am criticizing here is based on similarities regarding the original empirical *observations* (the explanandum).

Note that, in contrast to the stage approach, the resource account does provide some theoretical space for the consideration of individual differences already.
